# A 1-Dimensional Physiological Signal Prediction Method Based on Composite Feature Preprocessing and Multi-Scale Modeling

**DOI:** 10.3390/s25216726

**Published:** 2025-11-03

**Authors:** Peiquan Chen, Jie Li, Bo Peng, Zhaohui Liu, Liang Zhou

**Affiliations:** 1 Xi’an Institute Optics and Precision Mechanics, Chinese Academy of Sciences, No. 17 Xinxi Road, Xi’an 710119, China; chenpeiquan20@mails.ucas.ac.cn (P.C.); li-jie@mail.nwpu.edu.cn (J.L.); pengbo23@mails.ucas.ac.cn (B.P.); lzh@opt.ac.cn (Z.L.); 2 University of Chinese Academy of Sciences, Beijing 100049, China; 3 Key Laboratory of Space Precision Measurement Technology, Chinese Academy of Sciences, No. 17 Xinxi Road, Xi’an 710119, China

**Keywords:** physiological signal prediction, multiscale modeling, deep learning, composite feature matrix, convolutional neural network, long short-term memory, attention mechanisms, intracranial pressure, blood pressure, photoplethysmography

## Abstract

**Highlights:**

**What are the main findings?**

- A seven-dimensional data preprocessing technique based on physiological signal priors enhances cross-dataset prediction accuracy. Expanding the raw one-dimensional signal into (amplitude, width, rise/fall time, first/second derivatives, raw signal) dimensions significantly improves waveform prediction performance. For example, on the CHARIS dataset, CBAnet reduced RMSE and MAE by approximately 45% and 50%, respectively, compared to the suboptimal model, while R^2^ improved by around 39%.
- CBAnet achieves inter-waveform prediction by unifying local morphology and long-range dependencies. This design achieves the best overall RMSE/MAE/R^2^ metrics among BiLSTM, CNN-LSTM, Transformer, and Wave-U-Net, with significantly superior peak/phase fidelity and temporal consistency compared to other models. In training on the CHARIS dataset, CBAnet achieved an RMSE of 0.4903 and an R^2^ of 0.8451, surpassing the performance of other models.

**What are the implication of the main finding?**

- Advancing real-time non-invasive monitoring. CBAnet’s moderate parameter count and efficient inference enable continuous bedside/edge deployment as a viable solution for intracranial pressure/blood pressure waveform estimation.
- Cross-dataset generalization capability and clinical utility. Achieved consistent improvements across GBIT-ABP, CHARIS, and PPG-HAF datasets.

**Abstract:**

The real-time, precise monitoring of physiological signals such as intracranial pressure (ICP) and arterial blood pressure (BP) holds significant clinical importance. However, traditional methods like invasive ICP monitoring and invasive arterial blood pressure measurement present challenges including complex procedures, high infection risks, and difficulties in continuous measurement. Consequently, learning-based prediction utilizing observable signals (e.g., BP/pulse waves) has emerged as a crucial alternative approach. Existing models struggle to simultaneously capture multi-scale local features and long-range temporal dependencies, while their computational complexity remains prohibitively high for meeting real-time clinical demands. To address this, this paper proposes a physiological signal prediction method combining composite feature preprocessing with multiscale modeling. First, a seven-dimensional feature matrix is constructed based on physiological prior knowledge to enhance feature discriminative power and mitigate phase mismatch issues. Second, a network architecture CNN-LSTM-Attention (CBAnet), integrating multiscale convolutions, long short-term memory (LSTM), and attention mechanisms is designed to effectively capture both local waveform details and long-range temporal dependencies, thereby improving waveform prediction accuracy and temporal consistency. Experiments on GBIT-ABP, CHARIS, and our self-built PPG-HAF dataset show that CBAnet achieves competitive performance relative to bidirectional long short-term Memory (BiLSTM), convolutional neural network-long short-term memory network (CNN-LSTM), Transformer, and Wave-U-Net baselines across Root Mean Square Error (RMSE), Mean Absolute Error (MAE), and Coefficient of Determination (R^2^). This study provides a promising, efficient approach for non-invasive, continuous physiological parameter prediction.

## 1. Introduction

Real-time monitoring of physiological signals holds significant clinical importance. Through real-time monitoring systems, nursing staff or clinicians can continuously track patient conditions even when located in different rooms or floors, enabling swift intervention when conditions deteriorate. In intracranial pressure (ICP) management, continuous remote observation and timely alerts are particularly critical for preventing secondary brain injury. Recent studies indicate that wearable and environmental sensing technologies enable stable cross-spatial tracking, providing a vital technical foundation for clinical telemetry systems [1,2,3]. However, due to technical limitations of traditional methods, continuous and non-invasive measurement of certain critical vital signs remains challenging. Kamanditya et al. noted that patient motion artifacts significantly compromise the accuracy of blood pressure (BP) readings, particularly in settings requiring continuous monitoring such as intensive care units. Even minimal movement during measurement can introduce errors in blood pressure estimates when conventional methods are used. These motion-induced inaccuracies are especially pronounced in intensive care or prolonged monitoring environments, where patient activity is unavoidable and traditional methods often fail to account for such interference [4,5,6,7]. The gold standard for ICP monitoring involves inserting a pressure catheter into the ventricles. This invasive procedure carries high risks, is time-consuming, and may result in infection rates of 5–14% and bleeding rates of 5–7% [8,9,10,11]. Photoplethysmography (PPG) has been widely adopted as a non-invasive alternative for monitoring blood circulation. However, PPG waveforms obtained from different body locations exhibit variations in amplitude and morphology. For instance, fingertip PPG signals offer the highest quality, whereas signals from areas such as the forehead demonstrate smaller amplitudes and slightly different waveforms [12,13,14]. These factors all increase the difficulty of accurately predicting physiological signals.

In recent years, deep learning has provided new approaches to address the aforementioned challenges by predicting signals that are difficult to obtain directly through traditional methods, using readily accessible physiological indicators. Convolutional neural networks (CNN) excel at extracting local features, making them well-suited for processing detailed patterns in waveforms such as PPG [15]. Long Short-Term Memory (LSTM) networks excel at capturing temporal dependencies and demonstrate outstanding performance in processing time-series signals such as heartbeat sequences and blood pressure fluctuations [16,17]. Attention mechanisms can highlight key features within vast amounts of information, enhancing the model’s focus on crucial temporal segments [18]. Transformers are better at extracting spatiotemporal information [19]. Wave-U-Net bypasses traditional time-frequency transformations by directly processing raw time-domain waveforms, thereby better preserving signal details and phase information. This makes it more suitable for end-to-end learning waveform generation [20]. Many researchers have conducted studies on this topic, such as using optical PPG signals to estimate continuous blood pressure [21,22]. Alternatively, predicting ICP through BP measurements enables early warning of intracranial hypertension [11,23].

However, these methods currently have several limitations: many existing studies focus on predicting blood pressure values rather than actual waveforms, thereby losing the temporal characteristics of physiological signals. Furthermore, physiological signals vary even for the same event across different individuals or during different physiological stages of the same individual, making accurate prediction extremely challenging. Therefore, designing a high-precision fusion architecture to address the aforementioned issues is crucial. Research in many fields has also begun to employ multiple neural networks in parallel [24,25].

Based on the aforementioned motivations, this paper proposes the physiological signal prediction model CBAnet. Compared with existing methods, the innovations of this work are primarily reflected in the following three aspects. First, unlike traditional methods that rely solely on raw PPG waveforms, we introduce a composite feature construction method based on physiological signal priors, extracting more discriminative input representations from multiple dimensions such as waveform morphology and trend changes. Second, overcoming the limitations of single-architecture approaches, CBAnet integrates multi-scale convolutions, temporal modeling, and attention mechanisms to achieve a more refined characterization of waveform dynamics. Additionally, addressing the lack of concurrently collected head and fingertip PPG signals in existing public datasets, we supplement our analysis with the self-built dataset PPG-HAF. The main contributions are as follows:

(1) Proposing a data preprocessing method based on physiological signal priors. By integrating physiological signal features, it transforms one-dimensional signals into a 7-dimensional feature matrix, effectively enhancing training efficiency and accuracy. For instance, in the CHARIS dataset, CBAnet’s RMSE and MAE were reduced by approximately 45% and 50%, respectively, whilst the R^2^ improved by around 39%.
(2) Designed the high-precision fusion framework CBAnet, which unifies multi-scale local features with long-range dependency modeling. Attention mechanisms enable global reweighting and alignment, reducing errors in non-autoregressive end-to-end training while improving waveform morphology and phase fidelity. In the training results of the CHARIS dataset, CBAnet achieved an RMSE of 0.4903 and an R^2^ of 0.8451, outperforming other models.
(3) A real-time PPG acquisition system is established, alongside the head-finger synchronized dataset PPG-HAF, forming a complete acquisition-denoising-output workflow that provides high-quality data support for model training and evaluation.

The remainder of this paper is organized as follows. In Section 2, a brief review of relevant prior work on the methodology is provided. In Section 3, the materials and datasets employed in this study are introduced. In Section 4, the proposed methodology is elaborated on in detail. In Section 5, the experimental setup and results are presented, followed by an in-depth discussion in Section 6. Finally, Section 7 concludes the entire paper and highlights potential directions for future research.

## 2. Related Works

In recent years, deep learning techniques have revolutionized advances in physiological signal processing and medical prediction tasks. This section reviews deep learning-based research on physiological signal prediction, focusing on the evolution of model architectures from early single networks to complex hybrid models, whilst analyzing their contributions and limitations.

The inception of deep learning applications in physiological signal prediction can be traced back to researchers transferring established computer vision and natural language processing models to the medical domain. Early work aimed to utilize deep learning for the automatic extraction of features from raw signals—such as PPG and electrocardiography (ECG)—thereby replacing laborious manual feature engineering reliant on expert knowledge. For instance, Schlesinger et al. utilized the MIMIC-III database to explore methods combining CNNs with Siamese networks, offering novel approaches for achieving individualized calibration. These pioneering studies demonstrate the immense potential of data-driven methods in physiological signal modelling [26]. Early research predominantly focused on exploring the efficacy of single, mature deep learning architectures in predicting physiological signals. CNN were widely employed owing to their robust local feature extraction capabilities. Wang et al. proposed an end-to-end framework combining a CNN with a gated recurrent unit (GRU), which takes a single normalized PPG waveform as input and can simultaneously model local features and temporal dependencies [27]. Wang and Ji further demonstrated the potential of pure CNN models in blood pressure prediction, with the advantage of eliminating cumbersome manual feature extraction. However, the drawbacks of such pure CNN models lie in their reliance solely on local convolutions, which inadequately capture signals with extreme ranges, and their requirement for large-scale data to ensure generalization capability [28].

To more effectively model the temporal dependencies of physiological signals, Long Short-Term Memory (LSTM) networks and their variants have been introduced. Zhang et al. proposed a method employing bidirectional LSTMs (BiLSTM) combined with demographic information, demonstrating good accuracy across different cardiovascular disease cohorts [29]. However, the generalizability of this approach may be constrained by dataset scale and diversity. The U-Net architecture, with its encoder–decoder structure and skip connections, has demonstrated strong performance in signal waveform reconstruction tasks. Athaya and Choi pioneered the application of U-Net for non-invasive arterial blood pressure waveform estimation, demonstrating accuracy comparable to conventional cuff-based methods [30]. A limitation lies in the model’s potentially poor adaptability across different patients, with constrained robustness in complex clinical scenarios such as acute condition changes. Subsequent research, such as the Wave-U-Net model proposed by Lei et al., has successfully been employed to reconstruct intracranial pressure waveforms from arterial blood pressure signals, validating the architecture’s advantages in integrating multi-scale contextual information with local details [31]. In recent years, Transformer models have garnered attention for their robust global context capture capabilities. Ma et al. employed a Transformer architecture combined with knowledge distillation techniques, achieving excellent results in cuffless blood pressure estimation [32]. Ma et al. further innovated by employing a Transformer-based transfer learning approach, demonstrating robust performance across diverse datasets [33]. However, Transformer-based methods typically rely on substantial training datasets, and their transfer learning strategies impose stringent requirements on data quality and features, rendering their application to small-sample datasets challenging.

To overcome the limitations of single models, researchers have turned to hybrid models that combine the strengths of multiple networks and explore multimodal signal fusion. The fusion of CNNs and RNNs represents one of the most common and effective hybrid strategies, with CNNs responsible for spatial/morphological feature extraction and RNNs (such as LSTMs or GRUs) handling temporal modelling. Panwar et al. designed long-term recurrent convolutional networks for multi-task prediction [34]. Jeong and Lim employed CNN-LSTM for multi-task learning [35]. Zhang et al. achieved high standards in blood pressure estimation using a CNN-LSTM model, demonstrating excellent accuracy [36]. A drawback of such models lies in their substantial computational resource and data volume requirements. The BP-CRNN (Convolutional Recurrent Neural Network) proposed by Leitner et al. and the dual-stream CNN-LSTM architecture introduced by Shaikh and Forouzanfar further validate the hybrid paradigm’s advantages in feature extraction completeness and sequence modelling capability [37,38]. Attention mechanisms were introduced to enhance the model’s ability to focus on critical signal segments. El-Hajj and Kyriacou constructed a model incorporating bidirectional recurrent units and attention layers, significantly enhancing the stability and robustness of predictions [39]. Aguirre et al. proposed a framework based on a sequence-to-sequence architecture integrating attention mechanisms, directly converting PPG signals into blood pressure waveforms [40]. Xiang et al. proposed the McBP-Net model (based on CNN-LSTM) for multimodal signal fusion, demonstrating remarkable performance in dynamic blood pressure estimation [41]. However, its reliance on multiple sensors increases equipment costs and operational complexity. Tian et al. proposed the Parallel Convolutional and Transformer Network (PCTN), which employs dual branches to extract local and global features, respectively [42]. Their attention fusion module significantly enhances the capture of complex blood pressure fluctuation patterns. Zhu et al. introduced a multidimensional Transformer-LSTM-GRU fusion model for epilepsy prediction, offering novel insights for physiological signal forecasting [19]. The 1-D SENet-LSTM approach proposed by Gengjia Zhang et al. further enhances prediction accuracy by strengthening feature learning through a channel attention mechanism [43]. Research has also explored integrating deep learning with traditional machine learning approaches. Rastegar et al. proposed a hybrid model combining CNNs and Support Vector Regression (SVR), capable of automatically extracting features from ECG and PPG signals to achieve high-precision predictions. However, its computational complexity may limit real-time applications [44].

In summary, research into deep learning-based physiological signal prediction has evolved from the application of single models to complex, sophisticated hybrid architectures. As highlighted in reviews by Le et al. and Pilz et al., integrating physiological prior knowledge with deep learning represents a crucial future direction [45,46]. Although existing models have achieved remarkable results on specific datasets, they still face common challenges. These include the models’ generalization capabilities across different populations (such as the issues of signal quality and individual variation highlighted by Mejía-Mejía et al.), and their dependence on computational resources. These shortcomings represent the key points this study aims to address and improve [47].

## 3. Material

This paper utilizes one self-built dataset and two public datasets as experimental data. The following sections will provide detailed descriptions of the datasets and experimental settings.

### 3.1. Self-Built Dataset

**Figure 1:**
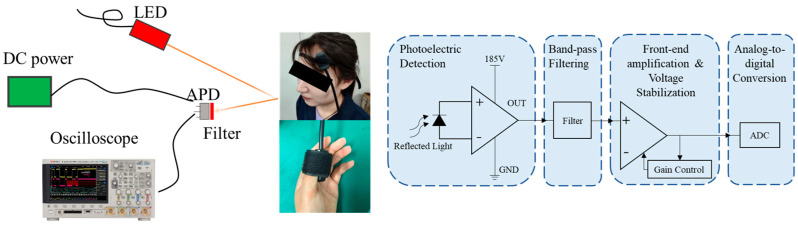
Signal Acquisition Schematic Diagram and Experimental Setup.

To capture a broader range of physiological signals, this paper designed a real-time PPG monitoring system that simultaneously acquires PPG signals from both the head and fingertip, establishing a dataset (PPG HAF) containing synchronized PPG signals from these two locations. The system principle is illustrated in Figure 1. The device comprises two independent photodetection units, fixed to the head and fingertip, respectively. Each detection unit incorporates an 850 nm central wavelength light-emitting diode (LED) light source and an avalanche photodiode (APD) detector, enabling non-invasive, continuous, and synchronous acquisition of PPG waveforms from both the head and fingertip. Signal acquisition is performed using a Keysight high-speed oscilloscope (Keysight, Santa Rosa, CA, USA) at a sampling frequency of 100,000 Hz.

Participants were healthy adult volunteers with no history of cardiovascular disease who had not recently consumed medications or alcohol that could affect physiological signals. All subjects received detailed explanations of the experimental procedures and signed informed consent forms. Prior to the experiment, participants rested quietly in comfortable chairs for at least 10 min to stabilize resting heart rate and circulatory status. Each experimental recording lasted approximately 20 s to obtain sufficient signal duration for subsequent analysis. Specifically, this study included 8 subjects; detailed demographic information for all participants is provided in Table A1. All experiments were conducted in a temperature-controlled dark room to ensure environmental consistency.

**Figure 2:**
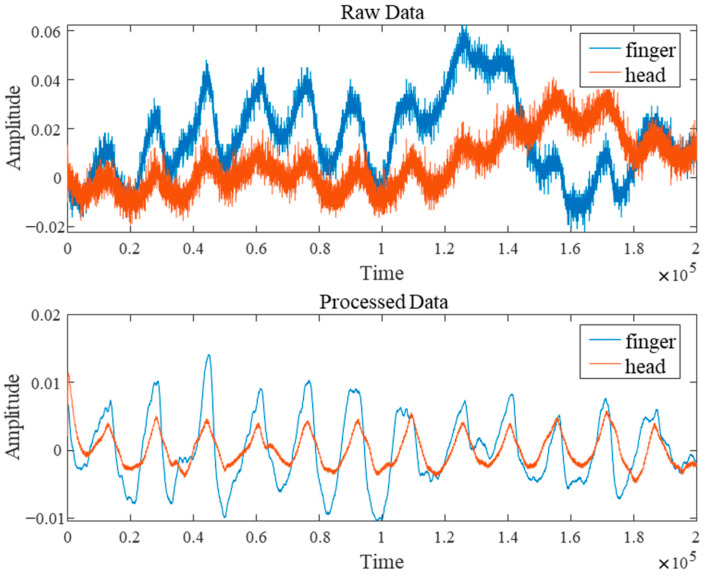
Comparison of Signal Processing Before and After.

After the experiment, the raw data underwent rigorous noise-reduction filtering preprocessing comprising three steps. First, a cubic spline interpolation method was employed to eliminate abrupt noise spikes caused by body movement or poor device contact. Second, morphological operations effectively removed baseline drift from the signals. Finally, a Butterworth filter (passband range approximately 0.5–10 Hz) was applied to suppress high-frequency electronic noise interference. Figure 2 shows a comparison of the raw signal before and after noise filtering. These preprocessing measures significantly improved the signal-to-noise ratio of the PPG signal, providing reliable data support for subsequent deep learning model training.

### 3.2. PublicData

To validate the model’s effectiveness, in addition to the self-built PPG HAF dataset, this paper also selected two public datasets. The first public dataset originates from an open database [48] (GBIT-ABP) that enables continuous cuffless monitoring of arterial blood pressure via a graphene bioimpedance electronic tattoo. This dataset comprises raw time series of four-channel bioimpedance signals, along with corresponding BP (sampled at 200 Hz) and PPG (sampled at 75 Hz) signals. This paper selected simultaneously recorded fingertip PPG and continuous blood pressure signals from this dataset to train and evaluate the model’s predictive capability for blood pressure waveforms.

The second set of publicly available data originates from the CHARIS database [49], which collects multi-channel physiological signal recordings from multiple patients diagnosed with traumatic brain injury (TBI), including ECG, blood pressure, and intracranial pressure. The acquisition system recorded signals from each channel at a sampling rate of 50 Hz, with high-frequency interference above 25 Hz filtered out. This study utilizes the simultaneously recorded BP and ICP waveform data from this database to evaluate the model’s ability to predict changes in intracranial pressure from arterial blood pressure waveforms.

### 3.3. Experimental Setup

All models undergo unified training and testing on the same dataset. The final 10,000 data points are reserved as the validation set, with the remaining data split into training and testing sets in an 8:2 ratio. Training samples were constructed using a sliding window approach with a window size of 700, stride of 100, and batch size of 32. This aims to select shorter time windows from longer sequences for end-to-end real-time prediction. Such sliding window processing effectively captures local signal features, ensuring the model can predict physiological data in real-time over brief intervals during practical applications. Input comprises preprocessed seven-channel physiological signals, with output being a one-dimensional physiological signal sequence matching the input length. The model employs Mean Squared Error Loss (MSELoss) as its objective function, utilizing the AdamW optimizer, with each dataset undergoing 100 epochs of training. All experiments were implemented within the PyTorch 2.6.0 framework using an NVIDIA RTX 4070 ti GPU.

## 4. Methods

Generally speaking, physiological signals exhibit pronounced continuity, with their values influenced not only by the current moment but also by coupling across multiple preceding and subsequent cardiac cycles, alongside low-frequency modulations such as respiration and autonomic nervous activity. Consequently, deep learning prediction faces significant challenges including strong nonlinearity, cross-periodic dependencies, inter-individual variability, and variable time lags (phase mismatch). We must simultaneously account for the relationship between local features such as rising edges and heavy beat notches and the overall low-frequency background, making accurate physiological signal prediction highly challenging.

**Figure 3:**
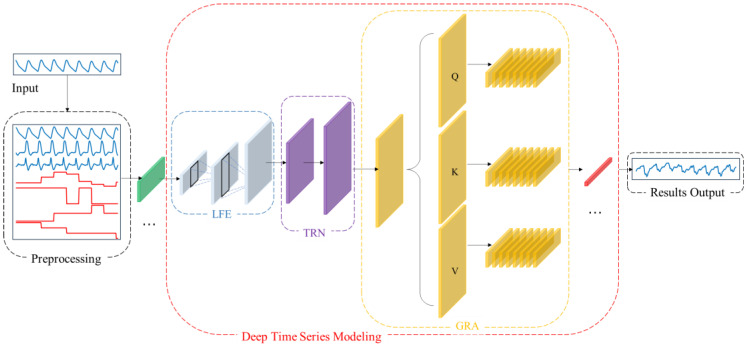
CBAnet Overall algorithm flow diagram.

To address the aforementioned challenges, this paper proposes CBAnet, an end-to-end prediction framework that integrates composite feature preprocessing with multi-scale modeling. The overall framework, illustrated in Figure 3, comprises three components: data preprocessing, deep temporal modeling, and result output.

First, data preprocessing is performed on the input side by introducing a 7-dimensional composite feature set based on physiological signal characteristics (raw waveform, amplitude, pulse width, rise time, fall time, first derivative, second derivative). This approach preserves the original information while incorporating prior features to reduce learning complexity and enhance computational efficiency.

Subsequently, one-dimensional convolutions are employed to extract multi-scale local features while suppressing noise. Bidirectional long-short-term networks are introduced to model long-term dependencies and sequential biases. Combined with multi-head self-attention, this approach redistributes information across positions and performs adaptive alignment throughout the entire time window. This highlights key segments and mitigates misalignment, balancing amplitude accuracy with dynamic consistency while enhancing robustness.

Finally, data integration and denormalization mapping achieve high-precision long-term regression prediction.

This design advances feature learning by incorporating prior features, thereby reducing parameter complexity and optimization difficulty. Concurrently, the synergy between multi-scale local feature extraction and long-term temporal modelling, coupled with the dynamic alignment mechanism of attention, significantly enhances prediction accuracy for complex nonlinear time series regression tasks.

### 4.1. Overview

Specifically, we denote the raw one-dimensional physiological signal as s. First, we perform data preprocessing on the raw signal s to obtain the aligned seven-channel feature matrix x. We then slice x into samples based on window length *T* and stride *S*, yielding $X_{i}$:

(1) $$X_{i}=x\left[t_{i}:t_{i}+T-1,:\right]\in {\mathbb{R}}^{T\times 7}$$

Inputting the processed sample $X_{i}$ into the network, our architecture comprises three sub-networks: local time-domain feature subnet $H_{1}=F_{\mathrm{LFE}}(x)$, long-term dependency representation subnet $H_{2}=F_{\mathrm{TRN}}(H_{1})$, and global reweighted alignment $Ƶ=A_{\mathrm{GRA}}(H_{2})$. Building upon this foundation, the attention-reweighted representation Γ is mapped to a prediction sequence through the time-step-by-step linear mapping module Ƶ, forming the overall inference architecture of the network:

(2) $$N_{\mathrm{CBA}}=\Gamma (A_{\mathrm{GRA}}\text{​}\circ \text{​}F_{\mathrm{TRN}}\text{​}\circ \text{​}F_{\mathrm{LFE}}),\;\hat{y}=N_{\mathrm{CBA}}(x)\in {\mathbb{R}}^{T\times 1}$$

Here, ∘ denotes function composition, and $\hat{y}$ represents the physiological signal sequence to be predicted, matching the input in length. By combining these three subnetworks, we achieve progressive modeling through a “local-global-alignment” approach: the convolutional layer focuses on fine-grained features like rising edges and reentrant notches; the bidirectional long-short-term memory module provides sequential and long-term memory priors; and the self-attention mechanism retrieves the most relevant evidence fragments globally at each time step. Experiments demonstrate that our composite architecture effectively mitigates individual variations, noise interference, and temporal mismatches.

During the training phase, mean squared error is employed as the primary loss function for end-to-end optimization of the network parameters θ:

(3) $$\theta^{*}=\arg\min_{\theta }\;L_{\text{MSE}}(N_{\mathrm{CBA}}^{\theta }(x),y)=\arg\;\min_{\theta }\frac{1}{T}\sum_{t=1}^{T}{\left(\hat{y_{t}}-y_{t}\right)}^{2}$$

Here, y denotes the actual physiological signal sequence to be predicted, while $N_{\mathrm{CBA}}^{\theta }$ represents the model with parameter θ. This objective balances local morphological fidelity with global trend consistency whilst maintaining controllable parameterization and training stability, thereby providing a structural foundation for subsequent analysis.

We present more details of our CBAnet in Algorithm 1:
**Algorithm 1**: CBAnet$\mathrm{Require}:\;\mathrm{An}\;\mathrm{initialized}\;\mathrm{network}\;N_{\mathrm{CBA}}$, a dataset D, window length T, step size S Step 1: Train the network 1: Data Preprocessing Perform preprocessing on the raw signal s to get the a seven-dimensional feature representation matrix x. $2:\;\mathrm{Perform}\;\mathrm{forward}\;\mathrm{computation}\;\mathrm{using}\;a\;\mathrm{sliding}\;\mathrm{window}\;\mathrm{with}\;\mathrm{step}\;\mathrm{size}:\;\hat{y}=N_{\mathrm{CBA}}(x)$ 3: Optimize network parameters θ$\;\mathrm{based}\;\mathrm{on}\;\mathrm{mean}\;\mathrm{square}\;\mathrm{error}:\;\theta \leftarrow \arg\min_{\theta }\;L_{MSE}$ 4: Repeat Until convergence Step 2: Process the prediction results 5: Obtain the prediction result for each size. 6: Read out linearly along step size to obtain the complete prediction result matching the original input signal length. 7: Inverse normalization and output the evaluation metrics (RMSE/MAE/R^2^). Output: 1-dimensional prediction results with the same length as the original input length.

### 4.2. Data Preprocessing

Previous studies have demonstrated significant temporal lags and phase shifts between different physiological signals [50], with substantial variations in heart rate and phase differences across subjects. This readily introduces inter-period mismatches [51,52,53] and generates annotation noise [54,55]. Therefore, to enhance model performance, preprocessing of the raw signal sequence based on cardiac cycle features is required prior to training. This aims to simultaneously preserve both periodic structure and transient dynamic information, thereby improving training speed and accuracy.

**Figure 4:**
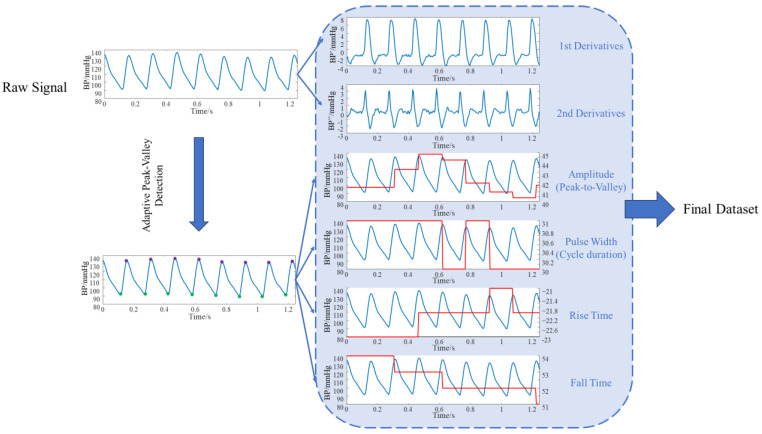
Data Preprocessing Flowchart.

Abay et al. employed features such as PPG signal amplitude, pulse width, and rise/fall times to predict intracranial pressure [56]. This demonstrates that these characteristics are closely correlated with intracranial pressure, hence we have selected the same features. Considering that these features reflect periodic variations in the time domain, we further introduced first-order and second-order derivatives as additional feature values. First, the entire sequence was segmented according to cardiac cycles. Four key temporal parameters were extracted from each cycle: amplitude (peak-to-trough, reflecting pulsatile intensity), pulse width (interval between adjacent peaks/troughs, reflecting cycle length), rise time (trough to peak, reflecting contraction dynamics), and fall time (peak to next trough, reflecting diastolic decay). Additionally, we computed first- and second-order derivatives at each sampling point. The first derivative characterizes the rate of change in the signal, while the second derivative captures curvature information, highlighting points of abrupt change. These parameters effectively characterize key features of the periodic motion inherent in physiological signals. The overall workflow is illustrated in Figure 4.

These feature values are extended to map to the sampling length of the original signal, forming a point-by-point aligned feature matrix alongside the original signal as input for downstream models. Specifically, the trough and peak positions of the i-th cycle of signal s are denoted as $t_{v,i},t_{p,i}$, while the boundary point of the i-th cycle is denoted as $t_{b,i}$. The specific feature values are shown in Table 1 below:

This preprocessing not only precisely characterizes the periodicity and dynamic features of signals along the time axis but also preserves information about both macro-level rhythms (periodic structures) and micro-level morphology (instantaneous derivatives). This enables the model to better capture the intrinsic patterns of signals during physiological signal prediction, facilitating stable convergence and improved generalization in subsequent learning, thereby achieving more accurate prediction results.

### 4.3. Deep Temporal Modeling Network

**Figure 5:**
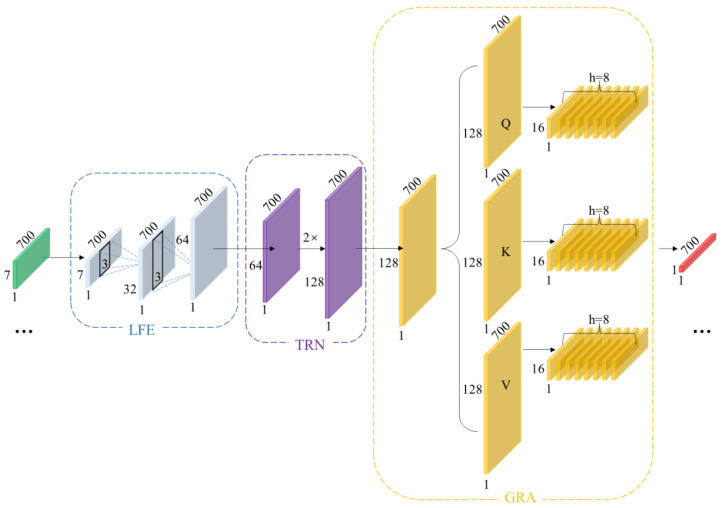
Schematic Diagram of Deep Temporal Modeling Network.

The schematic diagram of the deep temporal modeling network is shown in Figure 5, primarily divided into the following section: Local time-domain feature subnet (LFE), Long-Term Dependency Representation Subnet (TRN), and Global reweighting and alignment (GRN). The LFE aims to stably extract low-level features characterizing waveform geometric structures without sacrificing temporal resolution. To this end, this paper designs a two-layer lightweight feature extraction network LFE. The first layer can be understood as a combined approximation of the first-order local difference and smoothing of the input. The three-point neighborhood suppresses high-frequency random noise while preserving the local gradient information of rising/falling edges, as shown below:

(4) U1=ReLUx×W1+b1∈ℝT×32

The second layer performs nonlinear combinations while maintaining the sequence length, re-encoding “local geometric features” to enhance the distinctiveness of features at each time step. As shown below:

(5) HLFE=ReLUU1×W2+b2∈ℝT×64

To mitigate scale differences between features from diverse sources, this paper employs ReLU as the activation function. This ensures gradient stability while enhancing the separability of low-amplitude events. This high temporal fidelity strategy aligns closely with the critical discernment points of physiological waveforms. Consequently, without subsampling, we aggregate local neighborhood information using short kernel convolutions, suppressing high-frequency noise while preserving the waveform’s local geometric structure and phase information. Conversely, stride subsampling or pooling, while expanding receptive fields, tends to average out details and introduce phase errors. LFE amplifies small-scale geometric differences into discriminative features through its “short kernel-equal length-shallow layer” combination, providing morphologically stable inputs for subsequent processing.

Local convolutions alone cannot capture slow-varying modulations and variable time lags spanning extended periods. For instance, respiratory rhythms, changes in vascular compliance, and neurohumoral regulation exert slow, persistent effects on waveforms across multiple cardiac cycles. Furthermore, even identical events exhibit variable spatial locations across different individuals or physiological states within the same subject. Therefore, building upon features extracted from LFE, we stack bidirectional LSTM to form TRN architecture. As shown in the equation, let $H_{\mathrm{LFE}}\in {\mathbb{R}}^{T\times d}$, then:

(6) $$H_{t}=\left[\begin{array}{c} h_{t}^{\to };h_{t}^{\leftarrow } \end{array}\right]\in {\mathbb{R}}^{2h}$$

Capture causal dependencies forward (from past to present) and supplement counterfactual clues backward (from future to present); the combination yields a symmetric description of the present moment.

Among these, h represents the unidirectional hidden dimension, and bidirectional concatenation yields 2h dimension representation. Unlike fixed receptive fields achieved solely through convolution, TRN can better capture cross-period long-range dependencies without compromising temporal resolution.

Although TRN can already model longer time periods, it primarily relies on recursion between adjacent positions. In scenarios involving phase jitter, irregular periods, or rhythm drift, relying solely on recursion may introduce temporal step errors. To address this, we introduce an attention mechanism to construct the GRA network. This enables features from any two temporal steps within a sequence to be directly “visible” to each other, better aligning with the physical characteristics of temporal signals. As shown in the equation:

(7) $$Q=H_{\mathrm{TRN}}W_{Q},\;K=H_{\mathrm{TRN}}W_{K},\;V=H_{\mathrm{TRN}}W_{V}$$

In GRN, global soft alignment is achieved through attention weights normalized by softmax, which aggregates the most relevant temporal segments into the current representation via convex combination.

(8) $$\alpha =\mathrm{softmax}\text{​}(\frac{QK^{⊤}}{\sqrt{d_{k}}})\in \left[0,1\right]$$

(9) $$Ƶ=\mathrm{Concat}(\alpha V)W_{O}\in {\mathbb{R}}^{T\times 2h}$$

Here, $d_{k}$ represents the single-head dimension and $\alpha \in {\mathbb{R}}^{T\times T}$ denotes the attention weight. Through GRA, the model performs reweighting and soft alignment across the entire window, thereby mitigating amplitude and phase errors caused by cross-period mismatch and enhancing sensitivity to boundary moments.

### 4.4. Pointwise Regression Output

After completing global alignment, we employ a time-step-by-time-step linear readout to map the high-dimensional representation to target values. This approach preserves the interpretability of the upstream representation while minimizing parameters to reduce overfitting risk. The final output is obtained through the time-step-by-time-step linear mapping Γ:

(10) $$\hat{y}=\Gamma (Ƶ)=W_{out}{Ƶ}_{t}+b,t=1,\ldots ,T,$$

The loss function primarily employs mean squared error to ensure consistent amplitude at each point. Simultaneously, it incorporates weakly weighted shape and spectral consistency constraints to impose gentle limitations on slope and frequency band distribution, thereby preventing scenarios where similar amplitudes result in distorted shapes. As shown in the following equation:

(11) $$L=L_{\mathrm{MSE}}+\lambda_{\Delta }L_{\Delta }+\lambda_{\mathrm{PSD}}L_{\mathrm{PSD}}+\lambda_{\rho }L_{\rho }$$

Among these, $L_{\Delta }=\sum {\left(\Delta {\hat{y}}_{t}-\Delta y_{t}\right)}^{2}$ represents the first-order difference loss, $\lambda_{\Delta }$ denotes the weight, $L_{\mathrm{PSD}}={||\mathrm{PSD}(\hat{y})-\mathrm{PSD}(y)||}_{2}^{2}$ signifies the spectral consistency loss, $\lambda_{\mathrm{PSD}}$ indicates the weight, $L_{\rho }=1-\rho \left(\hat{y},y\right)$ denotes the correlation term, and $\lambda_{\rho }$ represents the weight.

Both training and inference employ a sliding window strategy with fixed window length: during training, a fixed window slides across long sequences to prevent information leakage. During inference, overlapping regions undergo smooth fusion to ensure the concatenated prediction sequence is strictly aligned with the ground truth sequence along the temporal axis.

### 4.5. Implementation Details

To validate the effectiveness of the proposed model, we compare it with four commonly used models: BiLSTM [57], CNN-LSTM [4], Transformer [22], and Wave-U-Net [58]. BiLSTM employs bidirectional recurrent layers to simultaneously model past and future dependencies in the temporal dimension, representing a classic sequence modeling approach. It is particularly effective at capturing rhythmic information such as the morphology of cardiac cycles and respiratory modulation. CNN-LSTM first extracts local features (e.g., wave peaks, rising edges) via front-end convolutions, then aggregates global temporal patterns through long-short term memory networks, enabling better utilization of local feature characteristics than pure LSTM. Transformers establish long-range dependencies through self-attention, making them better suited for modeling correlations spanning multiple cardiac cycles. Wave-U-Net employs a multi-scale encoder-encoder architecture to achieve end-to-end “waveform-to-waveform” mapping, simultaneously capturing local transients and global trends, making it ideal for end-to-end modeling of physiological signals.

To evaluate the performance of different methods in predicting physiological signals, this paper selected three commonly used evaluation metrics: Root Mean Square Error (RMSE), Mean Absolute Error (MAE), and Coefficient of Determination (R^2^). These metrics measure the deviation between model predictions and actual values, as well as the model’s goodness of fit, from different perspectives. They are widely used in evaluating the effectiveness of time series forecasting models.

1. RMSE is defined as the square root of the average of the squared deviations between the predicted value and the true value. Its mathematical expression is as follows, reflecting the average level of deviation between the predicted value and the true value.

(12) $$\mathrm{RMSE}=\sqrt{\frac{1}{n}\sum_{i=1}^{n}{\left(y_{i}-{\hat{y}}_{i}\right)}^{2}}$$

2. MAE is defined as the average of the absolute values of prediction errors, with the following mathematical expression. It measures the average magnitude of the error between the model’s predicted values and the actual values.

(13) $$\mathrm{MAE}=\frac{1}{n}\sum_{i=1}^{n}\left|y_{i}-{\hat{y}}_{i}\right|$$

3. The R^2^ measures the proportion of variance in the dependent variable explained by the model. It is commonly used to assess the goodness of fit of a regression model. Its formula is defined as the ratio of the sum of squares of actual values to the sum of squares of predicted values, as follows.

(14) $$R^{2}=1-\frac{\sum_{i=1}^{n}{\left(y_{i}-{\hat{y}}_{i}\right)}^{2}}{\sum_{i=1}^{n}{\left(y_{i}-\bar{y}\right)}^{2}}$$

Since physiological signal prediction constitutes a typical regression problem, we employ RMSE and MAE to quantify the magnitude deviation between model predictions and actual values, while using R^2^ to measure the model’s ability to fit the trend of the target signal. In prediction tasks such as continuous blood pressure or intracranial pressure monitoring, these metrics provide an intuitive reflection of the model’s error magnitude and reliability, serving as common standards for evaluating algorithm performance in relevant fields.

## 5. Experiment Results

Training the three datasets using the aforementioned method yielded the following results.

### 5.1. Data Preprocessing Results

To validate the necessity of data preprocessing, we conducted experiments on three datasets (PPG HAF, GBIT-ABP, and CHARIS) using CBAnet and a baseline model. We input both unprocessed and preprocessed data. We define:

(15) $$\Delta \mathrm{RMSE}\%=100\times \frac{{\mathrm{RMSE}}_{s}-{\mathrm{RMSE}}_{x}}{{\mathrm{RMSE}}_{s}}$$

(16) $$\Delta \mathrm{MAE}\%=100\times \frac{{\mathrm{MAE}}_{s}-{\mathrm{MAE}}_{x}}{{\mathrm{MAE}}_{s}}$$

(17) $$\Delta R^{2}=R_{s}^{2}-R_{x}^{2}$$

Table 2 summarizes the prediction performance of five models across three datasets, comparing results between raw data s and preprocessed data x. Experimental results demonstrate that data preprocessing effectively enhances the performance of all five model types. Post-preprocessing models exhibit consistent reductions in RMSE and MAE alongside sustained increases in R^2^, with CBAnet delivering particularly outstanding results on the CHARIS dataset: RMSE decreased by 45.9%, MAE by 50.3%, and R^2^ rose to 0.9345. These findings indicate that preprocessing mitigates phase mismatch and amplitude drift issues by explicitly extracting morphological and trend features related to the cardiac cycle. This enables models to simultaneously capture both local structural details and low-frequency background information, thereby significantly enhancing their generalization capabilities.

### 5.2. Five-Fold Cross-Validation

**Figure 6:**
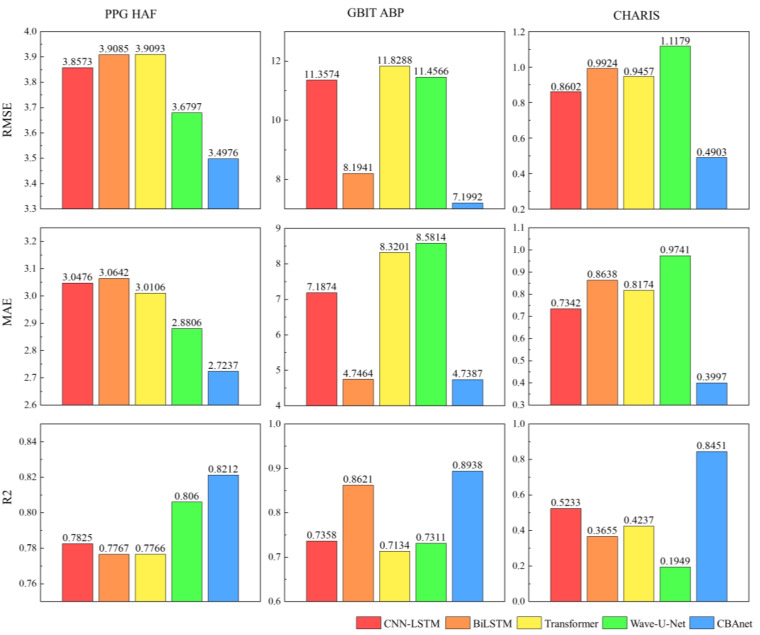
Bar charts of RMSE, MAE, and R^2^ parameters for five networks across three datasets.

**Figure 7:**
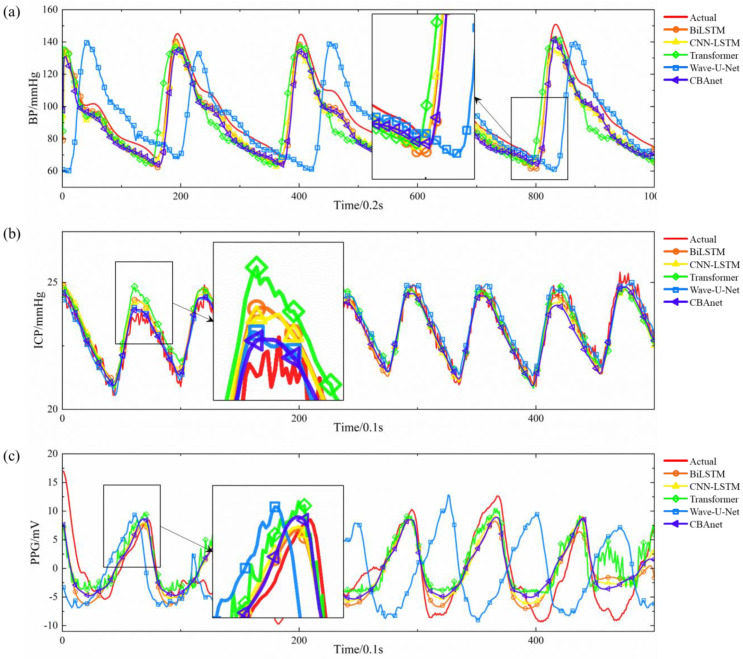
Comparison of actual and predicted signals across three datasets: (**a**) GBIT-ABP, (**b**) CHARIS, (**c**) PPG HAF.

To enhance the model’s generalization capabilities and achieve more accurate results, this section employs five-fold cross-validation for dataset training. By averaging the results across each fold, a stable assessment of the model’s overall performance is obtained. Subsequently, the optimal parameters derived from training were applied to predict values for a pre-segmented validation set comprising 10,000 points. This ultimately yielded the comprehensive training outcomes for each model. Partial results are shown in Table 3 below; the complete results can be found in the Table A2:

Experimental results across three datasets demonstrate that CBAnet achieves optimal performance. Specifically, on the PPG-HAF dataset, CBAnet achieves a validation set RMSE of 3.4976 and an R^2^ of 0.8212, outperforming all comparison models. Wave-U-Net and CNN-LSTM followed, while Transformer slightly underperformed CBAnet across multiple metrics. On the GBIT-ABP dataset, CBAnet achieved the best validation RMSE of 7.1992 and R^2^ of 0.8938. BiLSTM and Wave-U-Net yielded relatively good results, but CNN-LSTM and Transformer exhibited significant errors. On the CHARIS dataset, CBAnet again demonstrated outstanding performance, achieving a validation set RMSE of 0.4903 and an R^2^ of 0.8451, comprehensively outperforming all other models. Combining the validation set metric comparisons shown in Figure 6 and Figure 7 with the visualized prediction waveforms, CBAnet demonstrates superior signal fitting capability and stability across all datasets. Its prediction curves closely match the true values. Based on comprehensive evaluation metrics, CBAnet exhibits a clear advantage in the blood pressure waveform reconstruction task.

In contrast, the CNN-LSTM model exhibits a drawback of elevated peak amplitude in its prediction curve. This stems from the limited expressive capability of convolutions in capturing sharp points under subsampling and fixed receptive fields. While BiLSTM leverages bidirectional long short-term memory to capture both forward and backward temporal information, it lacks the detailed extraction capabilities of convolutions, resulting in slight deficiencies in reconstructing subtle waveforms. Transformers, as a popular recent architecture for sequence modeling, excel at capturing long-range dependencies. However, they inadequately represent local details during training, leading to slightly elevated peaks. This may stem from their reliance on large datasets and reduced sensitivity to minute morphological variations compared to local convolutions and recurrent memory structures. The Wave-U-Net model extracts multi-scale features through its encoder-encoder convolutional structure. However, the multi-layer sampling process causes partial loss of high-frequency detail information, resulting in insufficient output waveform detail. Additionally, this architecture lacks realistic temporal sequence modeling, exhibits significant deficiencies in time alignment, and suffers from slow inference speeds, making it unsuitable for real-time deployment.

### 5.3. Bland–Altman Analysis

**Figure 8:**
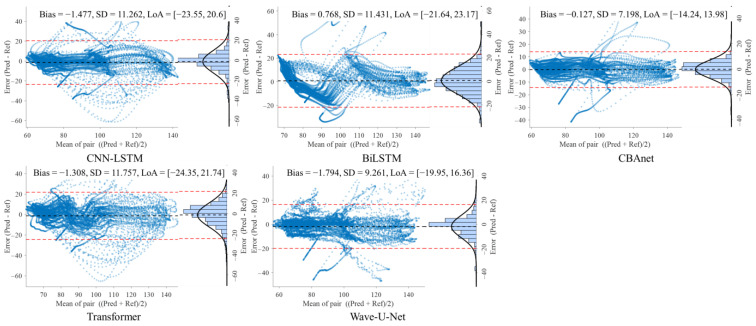
Bland–Altman analysis on the GBIT ABP dataset for five networks.

**Figure 9:**
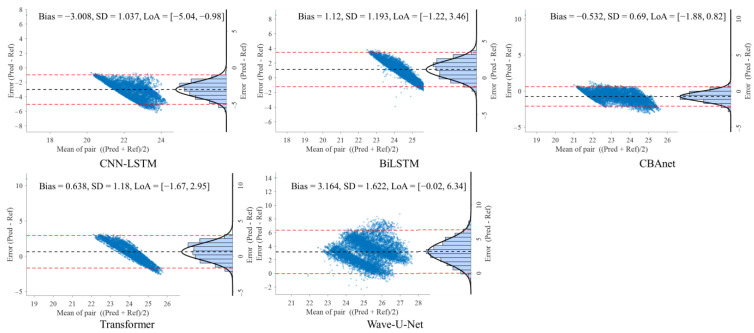
Bland–Altman analysis on the CHARIS dataset for five networks.

**Figure 10:**
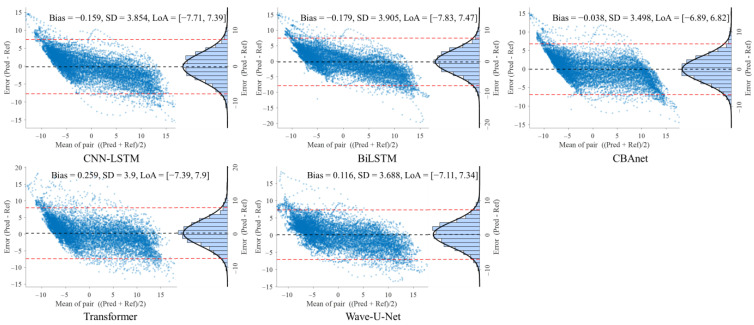
Bland–Altman analysis on the PPG HAF dataset for five networks.

To further evaluate the consistency between model predictions and reference signals, this study conducted Bland–Altman analyses on three datasets: GBIT-ABP, CHARIS, and PPG-HAF. Systematic bias and residual dispersion were quantified by calculating the mean bias and 95% limits of agreement (LoA), as shown in Table 4, Table 5 and Table 6. Figure 8, Figure 9 and Figure 10 present the corresponding scatter plots and error histograms, illustrating the distribution of differences between predicted and actual values [59].

Results show that on the GBIT-ABP dataset, CBAnet exhibits the narrowest consistency interval and smallest bias, with bias = −0.127 mmHg, SD = 7.2 mmHg, and a 95% LoA of [−14.24, 13.98] mmHg. In contrast, CNN-LSTM and BiLSTM exhibited larger consistency intervals of approximately ±22 mmHg with greater variability.

On the CHARIS dataset, CBAnet again demonstrated optimal performance with bias = −0.532 mmHg, SD = 0.69 mmHg, and a 95% LoA of [−1.88, 0.82] mmHg—the narrowest among all models. In contrast, Wave-U-Net and CNN-LSTM exhibited larger biases and asymmetric distributions, while CBAnet’s error points were uniformly distributed around zero with no significant proportional bias.

On the PPG-HAF dataset, CBAnet similarly maintained the smallest bias (bias = −0.038 mV) and narrowest consistency interval ([−6.89, 6.82] mV), with error distribution approximating normal and concentrated near zero; other models generally exhibited error ranges exceeding ±7 mV, featuring broader and more skewed distributions.

In summary, CBAnet demonstrated the smallest bias, smallest standard deviation (SD), and narrowest consistency limits across all three datasets, indicating its predictions closely align with actual signals. These results validate CBAnet’s capability to minimize systematic errors and its cross-scenario stability, establishing a reliable foundation for its clinical application in non-invasive physiological signal estimation.

CBAnet combines convolutional operations for detail extraction, bidirectional capture of long-term dependencies, and attention mechanisms focused on critical time points, effectively integrating local and global features. Its moderate parameter size enables more efficient training and facilitates robust generalization under limited data conditions. As a result, CBAnet balances waveform details with overall trends, producing prediction curves that closely match actual curves. Its error metrics significantly outperform other models. For instance, on the CHARIS dataset, CBAnet reduces RMSE and MAE by approximately 45% and 50%, respectively, compared to the next-best model, while increasing R^2^ by about 39%, demonstrating substantial accuracy improvements. From an engineering and clinical usability perspective, CBAnet exhibits strong robustness, balancing waveform details with global trends to maintain stable outputs across diverse physiological signals. It also offers good interpretability, with visualizable attention weights aiding result interpretation and anomaly localization. Deployment-friendly with moderate computational overhead, it facilitates real-time applications.

## 6. Discussion

The proposed method combining composite feature preprocessing with multiscale modeling demonstrates favorable performance in end-to-end modeling and prediction of physiological signals such as intracranial pressure under the evaluated datasets and short-time window settings. First, composite feature preprocessing based on physiological prior knowledge significantly enhances model performance. We expanded the original one-dimensional waveform into a seven-dimensional feature matrix encompassing amplitude, pulse width, rise time, fall time, first-order derivative, and second-order derivative. This provides richer morphological and trend information from the input side. Experimental results indicate that introducing composite feature preprocessing significantly reduces model prediction errors, with decreases in both RMSE and MAE, and an increase in R^2^. This suggests that the preprocessing method enhances model learning efficiency and short-term prediction accuracy.

Secondly, the multi-scale modeling framework CBAnet designed in this paper can simultaneously capture both the local geometric details (such as steep rising edges, peaks, and troughs) and the overall low-frequency trends in BP/ICP waveforms. Across the three tested datasets, CBAnet achieved superior overall error and correlation metrics.

During the data processing phase of our self-built dataset, we employ cubic spline interpolation to eliminate spike noise caused by motion artifacts or poor sensor contact. Morphological filtering is then applied to remove baseline drift, followed by bandpass filtering at appropriate frequencies to suppress high-frequency electronic noise. These steps enhance the signal-to-noise ratio to a certain extent, contributing to the stability of model training and inference.

Despite the positive outcomes of this study, several limitations remain. Given the inherent challenges of non-invasive intracranial pressure measurement, overall accuracy remains constrained, and consistency with reference waveforms varies across different datasets. Furthermore, the current model has been validated using limited data from a single cohort, and experiments have focused on short-term monitoring. However, stability and accuracy during long-term monitoring (≥1 h) are critical. While model runtime indicates near-real-time processing potential, additional validation is required before clinical application—particularly for extended recording durations, diverse cohorts, and consistency analysis—to assess clinical applicability. Secondly, this study primarily addresses single signal prediction. Future research may explore multi-parameter cascade prediction frameworks, such as sequential prediction chains like “head PPG → fingertip PPG → blood pressure → intracranial pressure.” As high-quality, diverse datasets continue to accumulate, model architectures will be optimized to reduce dependence on specific data distributions. This will enhance prediction accuracy while further balancing model complexity and interpretability.

## 7. Conclusions

Physiological signals exhibit distinct continuity and cross-period coupling, while being influenced by low-frequency modulation, individual variations, and variable time delays. However, existing deep learning approaches struggle to simultaneously capture both local features—such as rising edges and beat notches—and the global consistency of low-frequency backgrounds. Therefore, addressing the clinical challenge of ‘inferring hard-to-measure indicators from easily accessible signals,’ this paper proposes CBAnet, a fusion architecture for end-to-end continuous prediction of physiological waveforms. First, a 7-dimensional composite feature preprocessing serves as the input gateway, extracting features relevant to physiological signal prediction: amplitude, pulse width, rise time, fall time, first derivative, and second derivative. This stage performs denoising and dimensionality expansion to reduce computational load. Subsequently, a local temporal feature extraction module extracts stable local pattern features across multiple scales, highlighting peak/troughs and rising/falling edges. Contextual dependencies are established by integrating short- and long-term dependency representation networks, proving particularly effective for targets with temporal delay characteristics. The global reweighting and alignment module employs multi-head self-attention to explicitly weight and align data within time windows, amplifying the contribution of segments containing sudden events (e.g., blood pressure surges, ICP spikes). Experimental results indicate that data augmentation preprocessing combined with signal features contributes to improving model accuracy and performance. Furthermore, across the three preprocessed datasets, CBAnet demonstrates overall superiority over traditional methods in metrics such as RMSE, MAE, and R^2^, highlighting its feasibility and potential value in end-to-end prediction.

## Figures and Tables

**Table 1:** Features obtained from preprocessing.

Feature Value	Expression
Amplitude	${\mathrm{Amp}}_{t}=s\left(t_{p,i}\right)-x\left(t_{v,i}\right)$
Pulse Width	${\mathrm{Width}}_{i}=t_{b,i+1}-t_{b,i}$
Rise Time	${\mathrm{Rise}}_{i}=t_{p,i}-t_{v,i}$
Fall Time	${\mathrm{Fall}}_{i}=t_{b,i+1}-t_{p,i}$
1st Derivatives	$s\prime \left(t\right)=\frac{ds}{dt}$
2nd Derivatives	$s\prime \prime =\frac{d^{2}s}{dt^{2}}$

**Table 2:** Parameters such as RMSE, MAE, and R^2^ for different networks across three datasets.

Method	GBIT-ABP		
	RMSE↓	MAE↓	R^2^↑
CNN-LSTM	13.4430→11.7901 (Δ12.3%↓)	10.9660→7.8004 (Δ28.9%↓)	0.6298→0.7153 (Δ0.0855↑)
BiLSTM	11.8083→9.2924 (Δ21.3%↓)	8.8092→6.6348 (Δ24.7%↓)	0.7144→0.8231 (Δ0.1087↑)
Transformer	14.6022→11.4816 (Δ21.4%↓)	11.8972→8.1394 (Δ31.6%↓)	0.5632→0.7300 (Δ0.1668↑)
WaveU-Net	8.6071→8.1182 (Δ5.6%↓)	5.3458→5.2633 (Δ1.5%↓)	0.8479→0.8647 (Δ0.0168↑)
CBAnet	7.6313→**7.3170** (Δ4.1%↓)	5.1210→**4.8944** (Δ4.4%↓)	0.8807→**0.8903** (Δ0.0096↑)
Method	**CHARIS**		
	RMSE↓	MAE↓	R^2^↑
CNN-LSTM	1.1258→1.1231 (Δ0.2%↓)	0.8419→0.9055 (Δ7%↓)	0.5776→0.5797 (Δ0.0021↑)
BiLSTM	1.1036→1.0149 (Δ8%↓)	0.8735→0.7878 (Δ9.8%↓)	0.5941→0.6567 (Δ0.0626↑)
Transformer	1.3277→1.0827 (Δ18.5%↓)	1.0327→0.8407 (Δ18%↓)	0.4125→0.6093 (Δ0.1968↑)
Wave-U-Net	1.0848→1.0689 (Δ1.4%↓)	0.8300→0.8260 (Δ0.5%↓)	0.6078→0.6192 (Δ0.0114↑)
CBAnet	0.8212→**0.4444** (Δ45.9%↓)	0.6647→**0.3302** (Δ50.3%↓)	0.6744→**0.9345** (Δ0.2601↑)
Method	**PPG HAF**		
	RMSE↓	MAE↓	R^2^↑
CNN-LSTM	3.7141→3.5004 (Δ5.8%↓)	2.8937→2.7138 (Δ6.2%↓)	0.7984→0.8209 (Δ0.0225↑)
BiLSTM	3.8464→3.7964 (Δ1.3%↓)	2.9776→2.9507 (Δ0.9%↓)	0.7837→0.7893 (Δ0.0056↑)
Transformer	4.3385→4.1490 (Δ4.4%↓)	3.3669→3.2095 (Δ4.6%↓)	0.7249→0.7484 (Δ0.0235↑)
WaveU-Net	3.6263→3.6188 (Δ0.2%↓)	2.8277→2.8181 (Δ0.3%↓)	0.8116→0.8124 (Δ0.0008↑)
CBAnet	3.7630→**3.4702** (Δ7.8%↓)	2.9294→**2.7060** (Δ7.6%↓)	0.7930→**0.8240** (Δ0.0310↑)

**Table 3:** The results of the validation sets obtained from the three datasets.

Method	GBIT ABP		
	RMSE	MAE	R^2^
CNN-LSTM	11.3574	7.1874	0.7358
BiLSTM	8.1941	4.7464	0.8621
Transformer	11.8288	8.3201	0.7134
WaveU-Net	11.4566	8.5814	0.7311
CBAnet	**7.1992**	**4.7387**	**0.8938**
Method	**CHARIS**		
	RMSE	MAE	R^2^
CNN-LSTM	0.8602	0.7342	0.5233
BiLSTM	0.9924	0.8638	0.3655
Transformer	0.9457	0.8174	0.4237
Wave-U-Net	1.1179	0.9741	0.1949
CBAnet	**0.4903**	**0.3997**	**0.8451**
Method	**PPG HAF**		
	RMSE↓	MAE↓	R^2^↑
CNN-LSTM	3.8573	3.0476	0.7825
BiLSTM	3.9085	3.0642	0.7767
Transformer	3.9093	3.0106	0.7766
Wave-U-Net	3.6797	2.8806	0.8060
CBAnet	**3.4976**	**2.7237**	**0.8212**

**Table 4:** Bland–Altman analysis of the GBIT ABP database under different networks.

Model	Bias	SD	LoA ≈ Bias ± 1.96·SD
CNN-LSTM	−1.477	11.262	[−23.55, 20.6]
BiLSTM	0.768	11.43	[−21.638, 23.173]
Transformer	−1.308	11.757	[−24.351, 21.735]
Wave-U-Net	−0.179	9.276	[−19.974, 16.388]
CBAnet	**−0.127**	**7.2**	**[−14.236, 13.982]**

**Table 5:** Bland–Altman analysis of the CHARIS database under different networks.

Model	Bias	SD	LoA ≈ Bias ± 1.96·SD
CNN-LSTM	−3.008	1.037	[−5.04, −0.98]
BiLSTM	1.121	1.193	[−1.22, 3.46]
Transformer	0.638	1.18	[−1.67, 2.95]
Wave-U-Net	3.164	1.622	[−0.015, 6.34]
CBAnet	**−0.532**	**0.69**	**[−** **1.88, 0.** **82]**

**Table 6:** Bland–Altman analysis of the PPG HAF database under different networks.

Model	Bias	SD	LoA ≈ Bias ± 1.96·SD
CNN-LSTM	−0.159	3.854	[−7.714, 7.395]
BiLSTM	−0.179	3.905	[−7.832, 7.474]
Transformer	0.259	3.901	[−7.386, 7.905]
Wave-U-Net	0.116	3.688	[−7.112, 7.344]
CBAnet	**−0.038**	**3.498**	**[−6.893, 6.818]**

## Data Availability

The data supporting the results of this study are available upon reasonable request from the corresponding author.

## Abbreviations

The following abbreviations are used in this manuscript:

BP	Blood Pressure
ICP	Intracranial Pressure
PPG	Photoplethysmography
CNN	Convolutional neural networks
LSTM<	Long Short-Term Memory
LFE	Local time-domain feature subnet
TRN	Long-Term Dependency Representation Subnet
GRN	Global reweighting and alignment
PPG HAF	PPG signals from the head and fingertip, constructing a dataset
LED	Light-emitting diode
RMSE	Root Mean Square Error
MAE	Mean Absolute Error
R^2^	Coefficient of Determination
TBI	Traumatic Brain Injury
ECG	Electrocardiogram
CBAnet	CNN-LSTM-Attention
APD	Avalanche photodiode

## Appendix A

**Table A1:** Detailed demographic information for all participants.

Number	Gender	Age
Participant 1	F	27
Participant 2	F	26
Participant 3	F	40
Participant 4	F	31
Participant 5	M	29
Participant 6	M	28
Participant 7	M	29
Participant 8	M	27

**Table A2:** Five-fold cross-validation results across three datasets.

Dataset: GBIT-ABP			
Five-Fold Cross	RMSE	MAE	R^2^
Model: CNN-LSTM			
1	19.6481	11.2642	0.3592
2	11.9563	8.5788	0.723
3	9.6708	6.5005	0.8061
4	17.6997	13.261	0.481
5	13.2958	7.9929	0.6468
Average	14.4541 ± 3.6871	9.5195 ± 2.4237	0.6032 ± 0.1624
Validation	11.3574	7.1874	0.7358
Model: BiLSTM			
1	11.1869	6.5369	0.7911
2	7.9683	5.5917	0.8804
3	6.7019	5.4797	0.9057
4	1.0542	0.7833	0.998
5	8.159	5.3139	0.8679
Average	9.1169 ± 1.9165	6.3587 ± 1.3264	0.8447 ± 0.0506
Validation	8.1941	4.7464	0.8621
Model: Transformer			
1	29.4893	13.2017	−0.4434
2	12.0207	8.1412	0.72
3	9.8083	6.563	0.8005
4	15.4696	10.8204	0.6036
5	14.4795	9.1956	0.5811
Average	16.2535 ± 6.9052	9.5844 ± 2.2793	0.4525 ± 0.4549
Validation	11.8288	8.3201	0.7134
Model: Wave-U-Net			
1	14.4539	7.755	0.6532
2	9.5356	7.1013	0.8238
3	6.8389	5.4155	0.903
4	15.7793	10.8239	0.5875
5	6.6259	4.9264	0.9123
Average	10.6467 ± 3.8142	7.2044 ± 2.0882	0.7760 ± 0.1324
Validation	11.4566	8.5814	0.7311
Model: CBAnet			
1	9.841	6.4277	0.8393
2	8.1247	5.6151	0.8721
3	5.9226	4.5294	0.9273
4	13.5471	10.7827	0.696
5	7.9829	4.9686	0.8727
Average	9.0837 ± 2.5542	6.4647 ± 2.2518	0.8415 ± 0.0780
Validation	7.1992	4.7387	0.8938
Dataset: CHARIS			
Five-fold cross	RMSE	MAE	R^2^
Model: CNN-LSTM			
1	0.6884	0.5566	0.7449
2	0.8496	0.6748	0.6084
3	0.6452	0.5259	0.7612
4	0.8183	0.6397	0.6291
5	0.7308	0.5585	0.7008
Average	0.7464 ± 0.0770	0.5911 ± 0.0563	0.6889 ± 0.0609
Validation	0.8602	0.7342	0.5233
Model: BiLSTM			
1	0.6942	0.5621	0.7406
2	0.9114	0.7264	0.5494
3	0.6605	0.5384	0.7498
4	0.9092	0.7166	0.5421
5	0.7988	0.6061	0.6425
Average	0.7948 ± 0.1048	0.6299 ± 0.0779	0.6448 ± 0.0893
Validation	0.9924	0.8638	0.3655
Model: Transformer			
1	0.7252	0.5811	0.7169
2	0.7781	0.6123	0.6715
3	0.6364	0.515	0.7677
4	0.8821	0.6926	0.5689
5	0.8384	0.6444	0.6061
Average	0.7721 ± 0.0862	0.6091 ± 0.0598	0.6662 ± 0.0721
Validation	0.9457	0.8174	0.4237
Model: Wave-U-Net			
1	0.7086	0.5642	0.7297
2	0.8938	0.7191	0.5666
3	0.5845	0.4716	0.804
4	0.8683	0.677	0.5823
5	0.698	0.5421	0.727
Average	0.75007 ± 0.1153	0.5948 ± 0.0907	0.6819 ± 0.0921
Validation	1.1179	0.9741	0.1949
Model: CBAnet			
1	0.6556	0.5174	0.7686
2	0.9657	0.734	0.4941
3	0.4608	0.3648	0.8782
4	0.6781	0.5422	0.7453
5	0.6674	0.5301	0.7504
Average	0.6855 ± 0.1614	0.5377 ± 0.1174	0.7273 ± 0.1363
Validation	0.4903	0.3997	0.8451
Dataset: PPG-HAF			
Five-fold cross	RMSE	MAE	R^2^
Model: CNN-LSTM			
1	2.6433	1.9519	0.8025
2	2.2626	1.7423	0.8763
3	3.4935	2.6785	0.2403
4	3.3659	2.4883	0.6842
5	3.9997	3.1093	0.7107
Average	3.2372 ± 0.5144	2.4356 ± 0.4440	0.6230 ± 0.1965
Validation	3.8573	3.0476	0.7825
Model: BiLSTM			
1	2.4171	1.819	0.8349
2	2.5687	1.889	0.7043
3	2.475	1.8614	0.6187
4	3.2477	2.441	0.706
5	4.0038	3.113	0.7101
Average	2.9424 ± 0.6089	2.2247 ± 0.4990	0.7148 ± 0.0691
Validation	3.9085	3.0642	0.7767
Model: Transformer			
1	2.7632	2.1046	0.7842
2	2.6901	2.0463	0.6757
3	2.9591	2.2824	0.4549
4	3.4361	2.5889	0.6709
5	4.3171	3.4096	0.6629
Average	3.2331 ± 0.6012	2.4864 ± 0.4988	0.6497 ± 0.1071
Validation	3.9093	3.0106	0.7766
Model: Wave-U-Net			
1	3.1245	2.4861	0.7256
2	2.445	1.8338	0.7326
3	2.8796	2.2751	0.4837
4	2.9414	2.261	0.7599
5	3.6587	2.8771	0.7574
Average	3.0099 ± 0.3937	2.3466 ± 0.3395	0.6919 ± 0.1049
Validation	3.6797	2.8806	0.806
Model: CBAnet			
1	2.808	2.1624	0.7771
2	2.5971	1.9673	0.6977
3	2.4858	1.9141	0.6153
4	3.4169	2.614	0.6746
5	3.7576	2.9494	0.7446
Average	3.0131 ± 0.4920	2.3215 ± 0.3992	0.7019 ± 0.0561
Validation	3.4976	2.7237	0.8212
